# Interactions between the Prophage 919TP and Its *Vibrio cholerae* Host: Implications of *gmd* Mutation for Phage Resistance, Cell Auto-Aggregation, and Motility

**DOI:** 10.3390/v13122342

**Published:** 2021-11-23

**Authors:** Na Li, Yigang Zeng, Bijie Hu, Tongyu Zhu, Sine Lo Svenningsen, Mathias Middelboe, Demeng Tan

**Affiliations:** 1 Zhongshan Hospital, Fudan University, Shanghai 200032, China; li.na2@zs-hospital.sh.cn (N.L.); hu.bijie@zs-hospital.sh.cn (B.H.); tyzhu@fudan.edu.cn (T.Z.); 2 Shanghai Public Health Clinical Center, Fudan University, Shanghai 201508, China; zengyigang@shphc.org.cn; 3 Department of Biology, University of Copenhagen, 2200 Copenhagen, Denmark; sls@bio.ku.dk

**Keywords:** *Vibrio cholerae*, prophage, phage-host interactions, phage receptor, O-antigen

## Abstract

Prophage 919TP is widely distributed among *Vibrio cholera* and is induced to produce free φ919TP phage particles. However, the interactions between prophage φ919TP, the induced phage particle, and its host remain unknown. In particular, phage resistance mechanisms and potential fitness trade-offs, resulting from phage resistance, are unresolved. In this study, we examined a prophage 919TP-deleted variant of *V. cholerae* and its interaction with a modified lytic variant of the induced prophage (φ919TP *cI*^-^). Specifically, the phage-resistant mutant was isolated by challenging a prophage-deleted variant with lytic phage φ919TP *cI*^-^. Further, the comparative genomic analysis of wild-type and φ919TP *cI^-^*-resistant mutant predicted that phage φ919TP *cI*^-^ selects for phage-resistant mutants harboring a mutation in key steps of lipopolysaccharide (LPS) O-antigen biosynthesis, causing a single-base-pair deletion in gene *gmd*. Our study showed that the *gmd*-mediated O-antigen defect can cause pleiotropic phenotypes, e.g., cell autoaggregation and reduced swarming motility, emphasizing the role of phage-driven diversification in *V. cholerae*. The developed approach assists in the identification of genetic determinants of host specificity and is used to explore the molecular mechanism underlying phage-host interactions. Our findings contribute to the understanding of prophage-facilitated horizontal gene transfer and emphasize the potential for developing new strategies to optimize the use of phages in bacterial pathogen control.

## 1. Introduction

*Vibrio cholerae*, the etiologic agent of the diarrheal disease cholera, is one of the most prevalent aquatic human pathogens and is responsible for disease and mortality in many countries [1]. Important virulence factors in *V. cholerae*, such as the cholera toxin (CTX) and Zonula occludens toxin (zot), are encoded by prophages [2], i.e., bacteriophage genomes that are integrated into the genome of the bacterial host or exist as an extrachromosomal plasmid. The interactions with bacteriophages are, therefore, important for the pathogenicity and dissemination of virulence in *V. cholerae*. The CTX and Zot toxins are carried by filamentous phages, which do not lyse their host cells upon propagation but are secreted through the host membrane in an infection process called a chronic cycle. However, *V. cholerae* also contains prophages that follow the lysogenic life cycle, where temperate phages are spontaneously induced, thereby killing their host.

Despite *V. cholerae* being a highly studied model bacterium and an important human pathogen, the role of several prophages and the corresponding free phage particles, such as phage φ919TP, remain to be experimentally characterized [3]. Genome sequencing shows that phage φ919TP is a lambda-like phage, carrying a CI repressor, which in phage lambda governs the lysis/lysogeny switch in the host cells. Specifically, when CI repressor concentration is high, it represses the development of the lytic life cycle of the prophage [4]. However, experimental evidence of its function in 919TP-prophages containing *V. cholerae* strains is lacking [3]. In addition, previous work suggested that lipopolysaccharide (LPS) plays an important role in adsorption and infection by the φ919TP phage particle [2]. Consequently, the prevention of phage adsorption to LPS by mutational changes in the receptor may be a defense strategy in *V. cholerae*. However, details of the resistance mechanisms against phage φ919TP remain to be elucidated [5]. LPS consists of lipid A, the core, and O-antigen. The O-antigen controls key phenotypic traits of *V. cholerae*, such as biofilm formation, motility, and virulence [6]. Moreover, it provides a rational basis for the classification of environmental and clinical isolates. Environmental *V. cholerae* isolates can fall into various O-antigen serogroups, while only the O1 and O139 serogroups are reported to be associated with cholera disease. Thus, the identification of specific LPS surface receptors for phage φ919TP, and the potential implications of resistance mutations in the receptor for bacterial functional properties, are important for understanding the role of this phage for *V. cholerae* functional and genetic diversity.

Important requirements for assessing phage receptors are to find a bacterial indicator strain that is sensitive to the induced prophage, an ability to produce infective phage particles, and successful selection for phage-resistant mutant bacteria. One limitation of this assay is that the isolation of phage-susceptible indicator strains for plaque assay has proved difficult and time-consuming. Moreover, upon infection, temperate phages can lyse the host cell and proliferate, or they can promote lysogenization and remain dormant as prophages, which makes it difficult to select bacterial mutants that are resistant to a temperate phage. However, the obstacle of finding efficient indicator strains can be circumvented by constructing a prophage-cured derivative of these strains, as well as an obligate lytic variant of the phage, to facilitate the enumeration of viable phages and the selection of phage-resistant mutants by plaque assays. Further, comparative genomics approaches can be applied as a useful tool for providing insights into genetic determinants with a signature for host specificity [7]. The development of a method that specifically characterizes prophage-host determinants would be of general value for the identification of genes involved in the adsorption process.

In this study, we use *V. cholerae* prophage-deleted variants to characterize phage φ919TP-host interactions, determine the mechanism of phage adsorption, and quantify the effect of phage-resistant mutations on motility and biofilm formation. Our results showed that phage-resistant mutants had a single nucleotide mutation in gene *gmd*, known as a representative O-antigen biosynthesis protein. Taken together, our finding suggests that prophage-driven diversification might alter host adaptive dynamics, with potential ecological and evolutionary implications.

## 2. Materials and Methods

### 2.1. Vibrio cholerae Strains, Bacteriophages, Plasmids, Oligos, and Growth Conditions

Bacterial strains, phages, and plasmids used in this study are listed in Tables S1 and S2. *V. cholerae* O1 El Tor strain Vc1 carries prophage 919TP, which can be spontaneously induced and is poorly propagated in the *V. cholerae* host strain SM6 [3]. Basic characterization of the induced phage φ919TP and its interactions with SM6 has been provided recently [3]. Both *V. cholerae* and *Escherichia coli* S17-1 were routinely grown in LB broth with aeration or on LB agar plates at 37 °C. Antibiotics were added, when appropriate, at the following concentrations: 25 µg mL^−1^ (for *E. coli*) and 5 µg mL^−1^ chloramphenicol (for *V. cholerae*).

### 2.2. Mapping of the Phage Attachment Site on the Vibrio Chromosome

Total genomic DNA of strain Vc1 was isolated from 15 mL mid-log phase cultures, according to the manufacturer’s protocol, using Wizard Genomic DNA Purification Kit (Promega, Madison, WI, USA). The DNA concentration and quality were measured using Nanodrop 2000 and 1.5% agarose gel. The gDNA was sequenced at Shanghai Human Genome (Shanghai, China) using the Pacific Biosciences 10-kb library preparation protocol.

**Figure 1:**
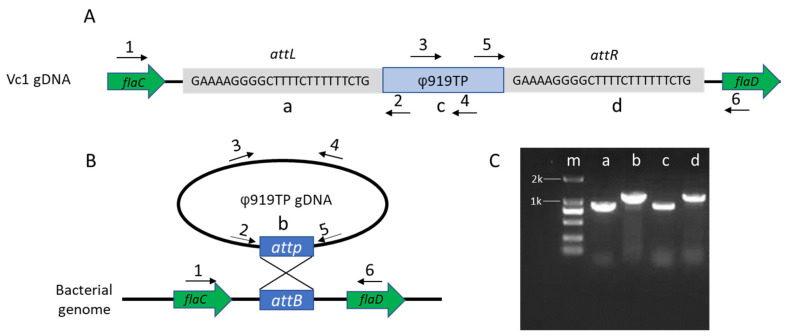
Characterization of prophage φ919TP attachment sites. (**A**) Schematic representation of the *attL* and *attR* sites between gene *flaC* and *flaD*. (**B**) Black arrows depict oligos used to characterize the excision of prophage 919TP PCR. Colony PCR analysis of prophage 919TP lysogeny of *V. cholerae* strain Vc1. (**C**) The resulting bands vary expectedly in size, depending on the primers used (lane m, DNA marker; lane a, primer 2 + 5, 934 bp; lane b, primer 1 + 2, 1031 bp, lane c, primer 3 + 4, 820 bp; lane d, primer 5 + 6, 1286 bp).

To determine the insertion site (*attL* and *attR*) of prophage 919TP in *V. cholerae* and evaluate whether prophage 919TP existed in a circularized form, circular consensus PCR was performed with primer 2 and primer 5, as shown in Figure 1, and using Vc1 genomic DNA as a template. The integration sites were further confirmed by sequencing (Sango, Shanghai, China) and aligned to the strain Vc1 genome using BLAST.

### 2.3. DNA Manipulations

**Figure 2:**
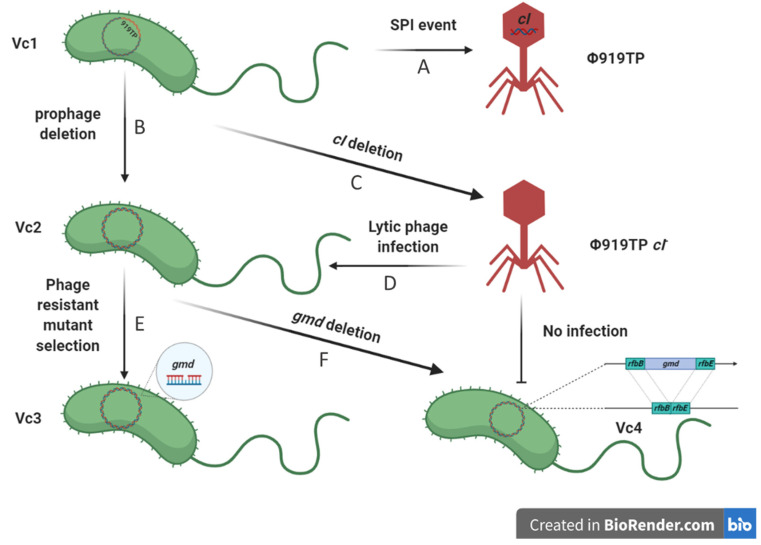
Schematic representation of phage receptor screen. Allelic exchange technologies by homologous recombination were used to test for host factors important in phage adsorption and resistance. (**A**) Prophage φ919TP undergoes a spontaneous prophage induction event (SPI) in the absence of external inducers. (**B**) In *V. cholerae* strain Vc1, we first generated a prophage 919TP-deleted strain Vc2 and (**C**) then a lytic φ919TP *cI*^-^ phage was constructed and (**D**) used to select for phage-resistant mutant Vc3 (**E**). Next, we performed a comparative genomic analysis of *V. cholerae* Vc1 and phage-resistant strain Vc3 to identify genetic determinants with a signature for host specificities. Site mutation (Vc3) or (**F**) deletion of gene *gmd* (Vc4) of bacterial host provided resistance to phage φ919TP *cI*^-^ infection. Figures were created using BioRender (https://biorender.com/ (accessed on 16 November 2021)).

Two mutants of *V. cholerae* O1 El Tor strain Vc1 were constructed, in which the prophage (Δ919TP) and potential phage receptor (Δ*gmd*), respectively, were deleted to investigate the effects on phage-host interactions (Figure 2). For the construction of the Δ919TP (denoted as Vc2) and Δ*gmd* (denoted as Vc4) mutants, the pDM4919TP and pDM4gmd plasmid were constructed, as described previously, using the primers listed in Table S2 [8]. Briefly, the flanking regions of the target gene were amplified, joined by PCR assembly, and cloned into pDM4. *E. coli* S17-1 λ-pir, harboring the resulting plasmid, was subsequently used to transfer the plasmid into *V. cholerae* Vc1 via bacterial conjugation. Transconjugants were selected on thiosulfate-citrate-bile salts sucrose (TCBS, Oxoid, Hampshire, UK) plates containing 5 µg mL^−1^ chloramphenicol, followed by recovery of deletion mutants on LB plates containing 5% (weight/volume) sucrose. Desired mutants were identified by PCR and verified by sequencing. Further analysis confirmed that the Δ919TP mutant did not produce infective φ919TP phage particles.

For the construction of a lytic variant of phage φ919TP (φ919TP *cI^-^*), the pDM4cI plasmid was constructed, as described above, with some modifications (Figure 2). The transconjugants selected on TCBS plates containing 5 µg mL^−1^ chloramphenicol were inoculated in LB broth containing 5% (weight/volume) sucrose and incubated at room temperature for 24 h; cell-free spent supernatant was collected and mixed with strain Vc2 for double-layer agar assay. Unmarked, double-crossover phage mutants with clear plaques were purified and verified by PCR and sequencing.

### 2.4. Isolation of Phage 919TP cI^-^-Resistant Variants

The prophage 919TP-deficient mutant *V. cholerae* strain Vc2 was used in a standard, double-layer plaque assay with the lytic phage φ919TP *cI^-^*, for the isolation of the phage-resistant mutant (Figure 2). Individual colonies were isolated from the center of plaques, after overnight incubation, and purified three times. Spot tests were performed to analyze their susceptibility to phage φ919TP *cI^-^*. Four phage-resistant derivatives of strain Vc2, with inherited resistant phenotype, were denoted as Vc3-A, Vc3-B, Vc3-C, and Vc3-D and selected for genome sequencing using Illumina Hiseq (Sangon, Shanghai, China), as described previously [9].

To elucidate the mechanism of phage-resistance, the assembled genomes of phage φ919TP *cI^-^*-resistant variants were aligned to the *V. cholerae* Vc1 reference genome, for comparative genomics, identification, and analysis of single nucleotide polymorphisms (SNPs) by GO categories, KEGG enrichment, and clustering analyses. Genes affected by high-impact SNPs were further verified by PCR and Sanger sequencing.

### 2.5. Adsorption Assays and Efficiency of Plating (EOP)

Briefly, overnight bacterial cultures of *V. cholerae* Vc2 and Vc3 strains were 1000-fold diluted in 10 mL LB broth and grown at 37 °C with aeration until OD_600_ was ~0.3. Phage φ919TP *cI*^-^ was added at a MOI of 0.00001. Samples were harvested every 2 min over a 10-minute adsorption assay by centrifugation at 16,000× *g* at 4 °C for 2 min, and the number of unadsorbed phages was quantified by plaque assay.

The EOP for phage φ919TP, φ919TP *cI*^-^, and KVP40 on each strain was determined by measurement of the number of plaque-forming units (PFUs) produced on individual hosts from a given stock, as quantified by spot test assay [10]. Briefly, aliquots of 2 µL phage stock were spotted on the lawns of the mid-log phase bacterial strains. After overnight incubation at 37 °C, the spots were quantified and the clarity was assessed as clear or turbid. The relative EOP of each individual strain was enumerated by using the following formula: the titer of target strain/the maximum titer of the original host strain. Each dilution was plated in triplicate.

### 2.6. Growth Curves of V. cholerae Strains

Bacterial strains were grown in LB broth overnight at 37 °C. Pre-cultures were 1000-fold diluted and inoculated in LB broth or sea salt buffer (Sigma-Aldrich, St. Louis, MO, USA) supplemented with 10% LB broth, respectively, to a final volume of 15 mL. The growth of strain Vc1, Vc2, Vc3, and Vc4 was determined by measuring optical density at 600 nm at 1-h intervals for 8 h.

### 2.7. Quantification of flaC and flaD mRNA Levels

Prophage 919TP is integrated between the *flaC* gene on one side and *flaD* gene on the other side. These two genes encode the subunit that polymerizes to form the filaments of bacterial flagella. To examine whether prophage deletion affected *flaC* and *flaD* gene expression, overnight cultures of *V. cholerae* strains (Vc1, Vc2, Vc3, and Vc4) were back-diluted 1:1000 in LB broth and grown at 37 °C, with 200 rpm agitation to an OD_600_ of ~0.8. Total RNA was extracted and reverse-transcribed using TRIzol™ Reagent (Thermo Fisher Scientific, Waltham, CA, USA) and RevertAid First Strand cDNA Synthesis Kit (Thermo Fisher Scientific, CA, USA), according to the manufacturer’s protocols [11]. The levels of *flaC* and *flaD* mRNA, relative to *hfq* mRNA (reference RNA), were determined by RT-qPCR, as described previously, using the primers listed in Table S2 [12]. Each experiment was independently repeated in triplicate with two biological duplicates.

### 2.8. Motility Assay and Crystal Violet Biofilm Formation Quantification Analysis

For the swarming motility assay, overnight bacterial cultures were 100-fold diluted and grown to a mid-log phase with an OD_600_ of ~1.0. Aliquots of 0.2 µL bacteria were spotted on soft LB agar (0.5%) and plates were incubated at 37 °C for 72 h for quantification of motility.

Biofilm formation was assayed in a 10-mL polystyrene tube with crystal violet staining, as described previously [13]. Briefly, overnight bacteria were 1000-fold diluted and inoculated in 5 mL LB broth for 10 days to establish a biofilm. After 10 days of inoculation, the liquid was removed, and the tubes were rinsed with SM buffer. Then, 6 mL 0.4% crystal violet (Sangon, Shanghai, China) was added to each tube and, after 15 min, the stain was removed. Tubes were washed with SM buffer to remove excess stain and left to dry for 5 min. A 6-mL volume of 33% acetic acid was added and left for 5 min to allow the stain to dissolve. The absorbance was measured at OD_595nm_.

### 2.9. Transmission Electron Microscopy (TEM)

Negative stain transmission electron microscopy was performed on exponentially growing cells, as previously described [14]. Briefly, overnight cultures were 1000-fold diluted in fresh LB broth and grown to the exponential phases. Bacterial cultures were fixed in a 2.5% glutaraldehyde solution (Sangon, Shanghai, China). Aliquots of 20 µL-drop culture were added to 200-mesh thick formvar/carbon film grids. After incubating at room temperature for 10 min, the grids were negatively stained with 2% phosphotungstic acid (SPI-chem, USA) for 3 min, and the remaining liquid was removed by filter paper. The grids were air-dried for 10 min and examined using an HT7800 transmission electron microscope (Hitachi, Tokyo, Japan).

### 2.10. Statistical Analysis

All statistics were performed using the student’s *t*-test (two-tailed) (OriginPro 9.6) to determine the statistical significance of the differences observed between strains. Differences were considered to be significant at *p* values of <0.05.

## 3. Results

### 3.1. Prophage Location and Integration Site in the V. cholerae Vc1 Genome

The genome of *V. cholerae* strain Vc1 contained an intact prophage 919TP and produced φ919TP phage particles at a low rate, known as spontaneous prophage induction (SPI) [3]. Due to ambiguities in the previous genome sequence, we could not identify the precise site for φ919TP integration. Therefore, we used the PacBio platform and circular PCR to determine the integration site in the Vc1 genome. Phage φ919TP, previously characterized as a *siphoviridae* phage, contains well-defined, conserved modules for DNA integration, regulation, DNA packing, head, and tail morphogenesis functions. The position and size of the prophage 919TP, in its respective genome, are illustrated in Figure 1A. Specifically, prophage φ919TP is integrated between the *flaC* and *flaD* genes, which encode flagellin C and flagellin D, respectively, involved in forming the filaments of bacterial flagella.

Next, to characterize phage excision and the regeneration of the target sequences termed *attB* (in the bacterial genome) and *attP* (on the phage genome), as well as the sites from the *attL* and *attR* junctions of the lysogen strain Vc1, we performed circular PCR and Sanger sequencing (Figure 1B,C). These assays showed that prophage φ919TP integrates into the *V. cholerae* Vc1 genome via recombination at the predicted *attP* site (GAAAAGGGGCTTTTCTTTTTTCTG) (Figure 1A and Figure S1).

### 3.2. Development of V. cholerae Vc1 as a Platform for Studying Prophage 919TP

**Figure 3:**
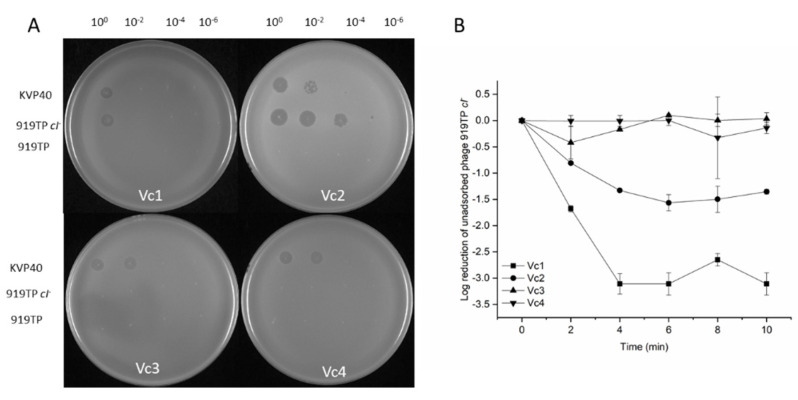
Phage sensitivity profiles of strains by spot test assay and adsorption assay. (**A**) Phage lysates (KVP40, φ919TP *cI*^-^, and φ919TP) were applied in 100-fold serial dilutions to the bacterial lawn of strain Vc1 (919TP lysogen), Vc2 (∆919TP), Vc3 (Vc2-phage φ919TP *cI^-^*-resistant mutant), and Vc4 (∆*gmd*); additionally, EOP was assessed. (**B**) Adsorption assay of phage φ919TP *cI*^-^ on strain Vc1, Vc2, Vc 3, and Vc4 plotted as the log of the percent of free phage in the cell-free spent supernatant. Unadsorbed free phages were determined as a ratio of free phage at the time point divided by the total phage added at the beginning of the assay. Results are representative of two independent experimental assays (*n* = 2).

Previous work has demonstrated that *V. cholerae* strains carry LPS mutations, which prevent the adsorption of phage φ919TP [3]. However, nothing is known about the interaction between the induced prophage and bacteria from population dynamics and evolutionary perspectives. It is often mentioned in the literature that prophages provide superinfection immunity to their related phages, through CI-repressor-based immunity and potentially other defense mechanisms [15]. Hence, we tested if strains that lacked prophages would be susceptible to prophage infection. To better understand the biological role of the prophage 919TP, we used a two-step, scarless–markerless system to delete the prophage 919TP [8], resulting in a prophage-free strain Vc2 (Figure 2B). The induced prophage φ919TP, obtained from Vc1, formed plaques on the strain lacking prophage 919TP, indicating that prophage 919TP excision from the chromosome is accompanied by loss of immunity to φ919TP infection. The plaques were turbid, rather than clear, confirming that both lytic and lysogenic developmental routes were available to φ919TP upon infection of Vc2 (Figure 3A). This allowed us to use the prophage-free mutant Vc2 as a phage-sensitive host for studying phage-host interactions.

### 3.3. The Function of Wild-Type cI-Like Repressor in Strain Vc1

The CI repressor protein, encoded by coliphage lambda, is known to act as a transcriptional repressor that allows lambda phage to establish and maintain lysogeny, preventing both phage DNA replication and excision from the bacterial chromosome [4]. According to the RAST (Rapid Annotation using Subsystem Technology) annotated genome analysis, prophage φ919TP carried a *cI*-like repressor gene, suggesting that it might also regulate lysogeny by CI-mediated transcriptional repression. Protein sequence alignment between CI repressors of prophage 919TP and phage K139 displayed 100% similarity, which has previously been shown to be required to maintain lysogeny of K139 phage in *V. cholerae* [16]. Therefore, we expected that the φ919TP *cI*^-^ would be unable to establish or maintain lysogeny in Vc2.

Using homologous recombination, we successfully removed the *cI*-like repressor open reading frame region (690 bp) from the prophage 919TP genome to generate a lytic phage (denoted as “φ919TP *cI*^-^”, see “Materials and Methods”) that grows only via the lytic cycle during infection of host cells (Figure 2C). A plaque assay experiment showed that phage φ919TP *cI^-^* gave clear plaques on the indicator strain Vc2, indicating failure to lysogenize. Both phage φ919TP and φ919TP *cI^-^* were able to form plaques on the prophage-cured derivative strain Vc2. However, the parental phage φ919TP produced very turbid (almost invisible from Figure 3A) plaques, whereas phage φ919TP *cI^-^* produced clear plaques (Figure 3A). Specifically, the indicator strain Vc2 displayed a higher susceptibility to phage φ919TP *cI^-^* than the wild-type phage φ919TP, while the parental strain Vc1 was only partially susceptible to the lytic phage φ919TP *cI*^-^ (5 orders of magnitude reduction in the efficiency of plating (EOP), compared to that of the strain Vc2) (Table 1) and was fully resistant to the parental phage φ919TP. In addition, a large number of infectious phage particles are produced by infection of the prophage-deficient strain Vc2 (~1 × 10^9^ PFU mL^−1^ ), while the wild-type strain Vc1 only produced 2 × 10^6^ PFU mL^−1^. Thus, the deletion of *cI* in the phage φ919TP generates true lytic development, resulting in phage progeny capable of forming clear plaques on strain Vc2.

### 3.4. Comparative Genomics and Functional Analysis of Phage φ919TP cI^-^ Resistant Mutant

Phage-resistant mutant strains derived from *V. cholerae* strain Vc2 were selected after exposure to phage φ919TP *cI^-^* (Figure 2D). Out of a total of 32 selection experiments, 8 resulted in the isolation of phage-resistant mutants. The resistant mutants were purified and screened by spot assay to confirm the inherited resistant phenotype. Of the eight resistant isolates, four were partially sensitive (i.e., reduced phage susceptibly when compared to the parental strain) after streak-purification, and four isolates displayed full resistance. The four fully resistant isolates were sequenced on an Illumina HiSeq platform, and assembled genomes were mapped to the *V. cholerae* strain Vc1 reference genome. One unique mutation in gene *gmd* was identified in one of the four isolates (strain Vc3, Figure 2E) and further verified by PCR and Sanger sequencing (Table S3). The gene *gmd* (GDP-mannose 4,6-dehydratase, EC: 4.2.1.47), which catalyzes the conversion of GDP-D-mannose to GDP-4-dehydro-6-deoxy-D-mannose, was predicted to be required for the biosynthesis of perosamine, which constitutes the backbone structure of O-antigen of the lipopolysaccharide in *V. cholerae* strain Vc1 [17]. The O-antigen is composed of 12 to 18 tetronate acylated perosamine repeats and plays important and various phenotypic roles, such as susceptibility to phages K139 and VP4 [18,19]. The full length of the GMD protein is 374 amino acids, and the single nucleotide deletion identified in isolate Vc3 results in the production of a truncated peptide of 105 amino acids, due to a premature stop codon.

### 3.5. Mutation in O-antigen Biosynthesis Gene gmd Suppresses the Phage Infection

To verify that the gene *gmd* was important for phage infection, the isogenic mutant Δ*gmd* (Vc4, Figure 2) was constructed and screened for EOPs, together with the wild-type strain and phage-resistant mutant. As expected, these in-frame deleted mutants were completely immune to phage φ919TP and 919TP *cI*^-^ infection (Figure 3), as a lawn of strain Vc3 and Vc4 did not support plaque formation (Figure 3A). In addition, the phage adsorption assay demonstrated that phage φ919TP *cI*^-^ had ~3-fold and ~1.5-fold reduced adsorption to the phage-resistant mutant Vc3 and Vc4 strains, compared to that of the wild-type strains Vc1 and Vc2, respectively, confirming that mutations in *gmd* blocked phage receptor interactions with phage φ919TP *cI*^-^ during the adsorption process (Figure 3B).

### 3.6. Prophage 919TP Provides Immunity to Phage KVP40

Prophages can mediate resistance to phage infection through various mechanisms. Little, however, is known about the range of phage-resistance, mediated by the prophage 919TP lysogens [20]. We, therefore, used vibriophage KVP40, which is known to use outer membrane protein K (OmpK) as its receptor [21], to assess the superinfection immunity among the strains Vc1, Vc2, Vc3, and Vc4 [11,21]. As seen in Figure 3A and Table 1, there is considerable variation in phage KVP40 infectivity among these four strains; specifically, the strain Vc1 displayed the lowest EOP of phage KVP40 among the 4 strains. By contrast, strains Vc3 and Vc4 became more susceptible to phage KVP40, compared to strains Vc1 and Vc2 (Table 1), likely because the absence of O-antigen makes the OmpK receptor more accessible for phage KVP40 adsorption [22]. Thus, the results suggest the presence of prophage 919TP and the functional *gmd* gene both reduce productive infections by phage KVP40 (Figure 3A).

### 3.7. The Absence of Gene gmd Reduces Motility and Affects Biofilm Formation

**Figure 4:**
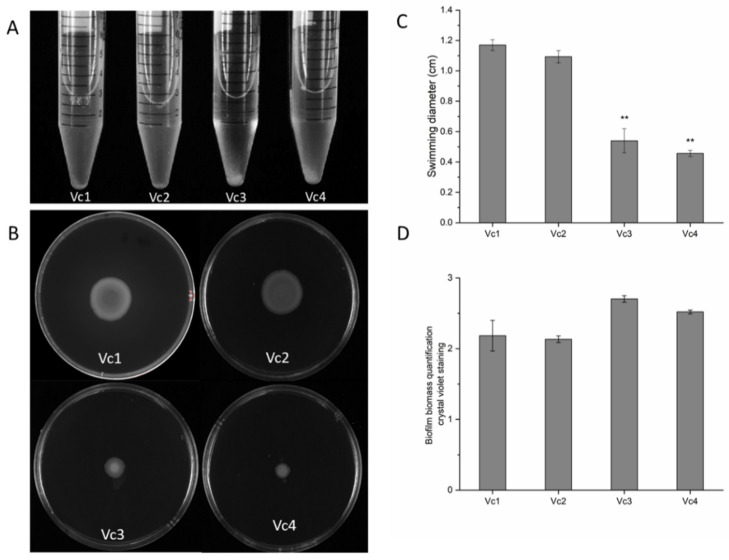
Roles of gene *gmd* in autoaggregation, motility, and biofilm formation. (**A**) *gmd* gene point mutation (Vc3) or deletion (Vc4) results in autoaggregation in liquid culture. Autoaggregation of wild-type and its derivatives was measured in stationary tubes after overnight incubation at 37 °C and was subsequently grown under static conditions for 2 h. (**B**) Examination of swarming motility of *V. cholerae* strains. Bacterial cells were inoculated at the center of 0.5% soft agar plates and incubated at 37 °C for 72 h. (**C**) Effect of deletion of the prophage 919TP and *gmd* gene on the swarming motility, as determined by swimming distance (diameter in cm) of the cells in motility agar. Quantification of swarm ring diameter of the wild-type Vc1 and the isogenic mutant strains (Vc2, Vc3, and Vc4) was performed. (**D**) Mutation of the *gmd* gene results in slightly enhanced biofilm formation on tube walls. Biofilm formation of wild-type and its derivatives was quantified by crystal violet after 10 days incubation under static conditions at 37 °C. The error bars represent the standard deviation, determined by t-test, in comparison to wild-type, ** *p* < 0.05. All experiments were carried out in triplicate.

We noticed that cultures of the phage φ919TP *cI^-^*-resistant mutant strain Vc3 auto-aggregated and settled at the bottom of the culture, whereas the wild-type Vc1 and prophage-deleted variant Vc2 cultures remained turbid under static culture growth conditions (Figure 4A). To examine any potential correlation between the loss of *gmd* and change in auto-aggregation and swarming motility patterns, we screened the wild-type Vc1 and its derivatives for their swarming abilities. *V. cholerae* strains Vc3 and Vc4 lacking *gmd* exhibited a significant decrease (*p* < 0.05) in motility in LB soft agar (0.5%), in comparison with strains Vc1 and Vc2 (Figure 4B,C).

Bacterial auto-aggregation is a key step in biofilm formation [23], and the quantification of biofilm formation in the parental strain and its derivatives over a long-term experiment revealed that, while biofilms of the Vc2 strain were similar to Vc1 biofilms, the *gmd* mutants Vc3 and Vc4 formed more biofilm than the wild-type, but not significantly different amounts (*p* > 0.05) (Figure 4D). In keeping with the auto-aggregation observed for the phage-resistant *gmd* mutant, these observations show that long-term biofilm formation is slightly stimulated in the absence of *gmd*. Together, these results suggest that loss of *gmd* limits motility and alters bacterial swarming behavior, as previously observed in *V. cholerae* [14].

**Figure 5:**
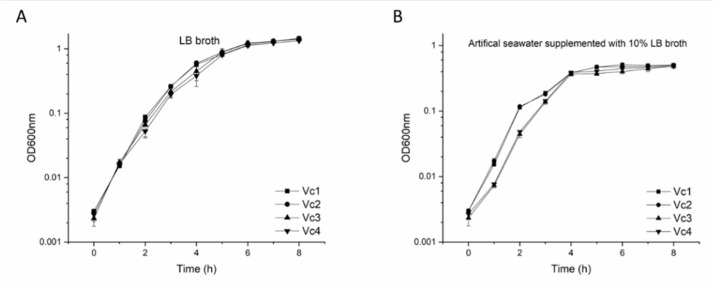
Growth of Vc1, Vc2, Vc3, and Vc4 in two different media: (**A**) growth in LB broth and (**B**) growth in artificial seawater containing 10% LB broth. Error bars represent data collected from three independent cultures with standard deviations. *X*-axis: time in hours; *Y*-axis: optical density (OD) at a wavelength of 600 nm.

We next assessed the overall effects of harboring prophage 919TP and the *gmd* gene on *V. cholerae* physiology. In doing so, we examined the growth rates and yields of wild-type strain Vc1 and its derivatives (Vc2, Vc3, and Vc4). All strains showed similar growth rates (~0.28 OD_600_ h^−^^1^) and grew to the same final yield in LB broth (Figure 5A), indicating that the absence of prophage 919TP and the product of the *gmd* gene had no measurable effect on growth in this medium. We also compared the growth of wild-type strain Vc1 and its derivatives (Vc2, Vc3, and Vc4) in a diluted nutrient liquid broth (artificial seawater supplemented with 10% LB), in order to mimic conditions that *V. cholerae* should encounter in the gastrointestinal tract or in estuaries (Figure 5B). As expected, dilution of the nutrients reduced the bacterial growth rates and bacterial yield, relative to LB broth. In the low-nutrient medium, the phage-resistant *gmd* mutant appeared to recover slightly slower than wild-type from the dilution of the stationary-phase overnight culture into the fresh medium in the first 2 h, with growth rates of ~0.055, ~0.057, ~0.021, and ~0.023 h^−1^ for strains Vc1, Vc2, Vc3, and Vc4, respectively; however, all four strains reached the same yield after 4 h.

**Figure 6:**
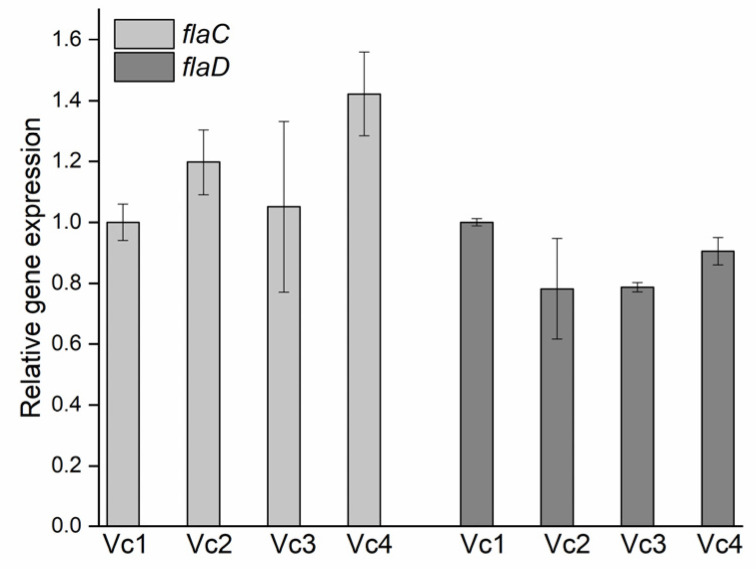
*flaC* and *flaD* mRNA levels of wild-type and its derivative mutants. Bacterial mRNA was isolated in (duplicate) from cells grown to an OD600 of 0.6 and quantified with gene-specific primers by reverse transcriptase quantitative PCR. “Relative mRNA expression” corresponds to the level of *flaC* and *flaD* mRNA after normalization to the level of *hfq* mRNA in the same sample. Error bars represent standard errors of the mean, with *n* = 2. All experiments were performed three times, with two biological duplicates.

**Figure 7:**
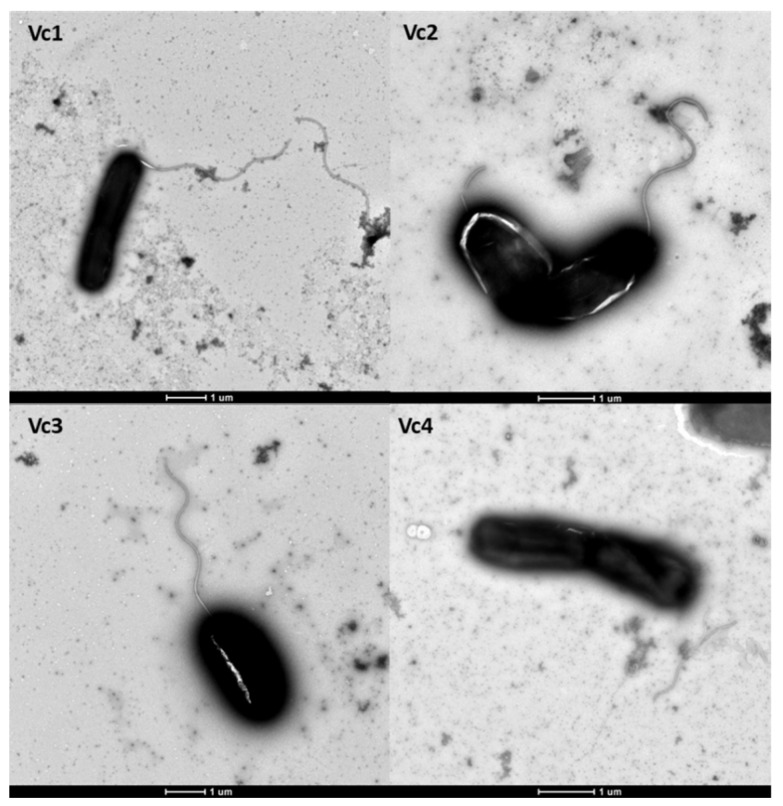
Transmission electron microscope images of *V. cholerae* wild-type Vc1 and mutant strains (Vc2, Vc3, and Vc4). Images were taken randomly from six different locations for each strain. One representative TEM image was selected to characterize the flagellar morphology. Bars, 1 µm.

*V. cholerae* is highly motile and possesses a flagellum sheathed in O-specific polysaccharide, and the motility of *V. cholerae* correlates with its ability to cause disease [24]. Since the intergenic region of flagellin subunit proteins FlaC and FlaD are disrupted by prophage 919TP, this prompted us to investigate whether the integration of prophage 919TP would affect the expression of *flaC* and *flaD*. Deletion of prophage 919TP caused no reduction (*p* > 0.05) in the relative *flaC* and *flaD* expression in the strain Vc2, relative to Vc1 (Figure 6), indicating that the excision of prophage 919TP does not affect flagellar gene expression. In addition, there was no difference in the level of gene expression of *flaC* and *flaD* among the three variants, Vc2, Vc3, and Vc4 (Figure 6), suggesting that the *gmd* gene product does not affect the expression of these flagellar genes. Further, analysis of flagella, using transmission electron microscopy (TEM), revealed that Vc2, Vc3, and Vc4 produce a single polar flagellum that is indistinguishable from that of the wild-type strain Vc1 (Figure 7). The observed detachment of flagella from the bacterial cells in Vc4 was likely due to the fixation, as previously reported [25]. Thus, the excision or integration of prophage 919TP does not prevent flagellum formation, as also verified by the swarming motility assay of the prophage-deleted strain Vc2 (see Figure 4B). The TEM images further demonstrate that the motility defect of the *gmd* mutants Vc3 and Vc4 is not due to an inability of the *gmd* mutant to synthesize flagella.

## 4. Discussion

The filamentous prophage CTXφ has been extensively studied, due to its role in the virulence of the human pathogen *V. cholerae* [26]. However, little is known about another prophage, φ919TP, and its potential roles in host evolution and functional properties, despite its widespread distribution among *V. cholerae* populations. Here, we show that O-antigen plays an important role, employed by pathogenic *V. cholerae*, in resisting φ919TP infection, which is the major component of the surface LPS; additionally, it plays a role in *V. cholerae* aggregation and biofilm formation.

### 4.1. Development of V. cholerae Vc1 as a Platform for Studying the Roles of Prophage 919TP

First, we developed a counter-selection system to delete prophage 919TP from the strain Vc1 bacterial chromosome, in order to generate a mutant strain, Vc2, which was susceptible to phage particles arising from the induced prophage. The functional properties of the prophage-free strain could then be compared to the wild-type strain Vc1. The prophage-deleted variant Vc2 was not affected, with respect to growth rate, motility, or biofilm formation phenotypes. Measurements of the *flaC* and *flaD* levels showed that prophage 919TP excision and integration did not affect the expression of neighboring genes. These results were interesting, as it has been suggested that retention of the prophage is a selective advantage, from the host perspective [27]. Previous work has shown that biofilm production can be strongly stimulated by the presence of prophages in the host genome, since spontaneous prophage induction strengthens the biofilm matrix by releasing various components, such as cell debris, eDNA, and polysaccharides [13]. However, recent *Proteobacteria* studies on engineered prophage-free strains have shown little difference between wild-type and prophage-deleted strains; additionally, the bacterial host in these cases can control phage promoters, enabling the host to silence unnecessary gene expression in the lysogenic life cycle of the phage [28,29]. It should be emphasized, however, that phenotypic differences, other than those measured here, may have been affected by the prophage.

Second, we constructed a lytic phage φ919TP *cI*^-^ mutant by deleting gene *cI* from the prophage 919TP genome. Comparative characterization of the prophage-free variant and wild-type strain, in multiple experiments, demonstrated that the sensitivity of strain Vc2 to phage φ919TP *cI^-^* depends on O-antigen biosynthesis. A common form of phage resistance is associated with the loss or altered structure of the receptors, which blocks phage adsorption [30]. The current study extends beyond the previous findings by demonstrating that phage φ919TP likely uses O-antigen for adsorption and that inactivation of the *gmd* gene, within the O-antigen gene cluster, encoding GDP-mannose 4,6-dehydratase, results in resistance against phage φ919TP. This observation supports the previous reports that O polysaccharide of the lipopolysaccharide can serve as a phage receptor in many gram-negative bacteria [31,32]. In some cases, more than one receptor may be required for phage adsorption and infection, as seen in *E. coli* phage T5, which uses the O-antigen’s polymannose moiety of the host LPS and outer membrane protein receptor FhuA together for irreversible attachment [33]. Here, we determined that the deletion of *gmd* in the strain Vc2 resulted in the loss of phage adsorption and host susceptibility to phage φ919TP *cI^-^*. The underlying mechanism by which phage φ919TP adsorbs to *V. cholerae* Vc1 and enters either lysogenic or lytic development is unknown. In particular, the molecular interactions between the host attachment protein(s) of the phage and O-antigen need to be elucidated.

### 4.2. Implications of Phage Resistance for Bacterial Physiological Properties

In general, phage infection pressure selects for receptor mutations in bacteria, with a cost to their fitness, making the mutants less competitive than their phage-susceptible counterparts in both environmental samples and infection models [34]. In the current study, we found that phage φ919TP *cI^-^*-resistant *gmd*-mutants influenced bacterial swarming motility. This is in agreement with a previous finding, in which a *waaL* mutant that lacks O-antigen also had impaired motility, affecting the ability of *V. fischeri* to spread in soft agar [35]. In addition, LPS-specific, antibody-mediated motility arrest, at the level of the individual *V. cholerae* bacterium, has been demonstrated to effectively inhibit cholera dispersal or provide protection in vivo [36]. O-antigen is considered a major virulence factor that is required, for instance, in vertical localization, small intestine colonization, and resistance to the immune response and antibiotics [32]. After surviving the acidic environment of the stomach, motility is then involved in the initial penetration of intestinal mucin, which overlays the intestinal epithelium [37,38]. Here, we show that interactions between phage φ919TP and *V. cholerae* influence the O-antigen and, thus, potentially, the virulence of the pathogen. Although the precise mechanism behind this phenotypic change is unknown, it was recently proposed in *Salmonella enterica* serovar Typhimurium that the Rcs phosphorelay can sense the defects in the structure of the LPS core and control flagellar gene expression [39]. A general explanation of our results is that *V. cholerae*’s single flagellum is covered in LPS, which displays O-specific polysaccharide (OSP). Once gene *gmd* is disrupted, *V. cholerae* motility is reduced, presumably due to the loss of the O-antigen. The loss of the O-antigen could ultimately deform its structure and inhibit its function. Therefore, overcoming the decreased force of the rotating flagella may be an obstacle to the bacteria. Other cell-surface determinants, such as type IV pili and fibrils, have been associated with social motility and cell–cell cohesion as well [40,41]. Determination of the complete composition, structure, and assembly of all of these cell-surface components, including the LPS, fibrils, and flagella, is essential to further our understanding of the relationship between the O-antigen and motility functions in *V. cholerae*.

Thus, our work adds to an increasing body of evidence that phage selective pressure is associated with certain fitness costs and that these trade-offs may affect virulence in *V. cholerae*. The broad host range of φ919TP *cI*^-^ suggests that it could have therapeutic potential for cholera infections. Thus, the strictly lytic life cycle of φ919TP *cI*^-^ would make this phage a good candidate for phage control of *V. cholerae*. Moreover, since lipopolysaccharide O1 antigen is a general target for bacteriophages, it could also be affected by other phages [3]. The use of genetically modified prophages has been suggested as a potential treatment strategy against bacterial infection. For instance, by using the bacteriophage recombineering of electroporated DNA (BRED) technique to precisely remove phage repressors, a synthetic lytic phage has been used for the treatment of a patient with a disseminated, drug-resistant *Mycobacterium abscessus* [42,43].

### 4.3. Effects of O-antigen Mutation on Biofilm Formation in V. cholerae

Bacterial auto-aggregation can be regarded as a self-recognition process in environmental and pathogenic isolates, and it is believed to play an essential role in the avoidance of the host immune system and other antimicrobial agents. Examples of both O-antigen mutation-activated and -repressed biofilm formation can be found in the literature. For instance, an *Actinobacillus pleuropneumoniae* O-antigen LPS mutant was previously shown to be deficient in biofilm formation, indicating the importance and significance of O polysaccharide expression in biofilm formation [44]. However, the phenotypes of φ919TP *cI^-^*-resistant isolate Vc3 and Δ*gmd* mutant Vc4 suggested the opposite scenario. Here, we show a slightly increasing (but not statistically significant) trend in biofilm formation in φ919TP *cI^-^*-resistant isolates, i.e., a strain mutated in the *gmd* gene and a Δ*gmd* mutant, suggesting that O-antigen might repress biofilm formation in *V. cholerae* Vc1. Our finding supports a previous study showing that the Δ*gmd* strain had an increased capacity to deposit biomass on an abiotic surface, relative to the WT strain [14]. Therefore, a large interspecies variation in O-antigen-mediated biofilm formation may exist.

## 5. Conclusions

Spontaneous prophage induction is an important, but often overlooked, phenomenon in bacterial populations, in the absence of external inducers [45]. Such events may result in horizontal gene transfer to other bacterial strains or species, as well as alter the relative abundance of the specific bacterial communities. In order to gain a better understanding of how spontaneous prophage activity drives the evolution of bacteria and the underlying molecular mechanisms, detailed knowledge of phage-host interaction is required. Here, we have described a simple method for the identification of possible phage receptors by mutagenesis and manipulation of *V. cholerae* and vibriophage genomes. This strategy takes advantage of a two-step homologous recombination process that allowed us to precisely remove the fragments from bacterial genome to yield seamless bacterial and prophage mutants. As a proof of concept, we successfully characterized the gene *gmd* involved in the phage φ919TP infection process. Further, we experimentally demonstrated the biological functions of the phage receptor-related gene, such as autoaggregation, motility, and biofilm formation. Together, this study demonstrates the power and novelty of applying precision-engineering to the *V. cholerae* genome for studying prophage-host interactions. Hopefully, these tools can be utilized to advance the potential application of phage therapy.

## Figures and Tables

**Table 1:** Host range of the phages: results of a spot test and relative efficiency of plating (EOP), EOP is calculated as the PFU mL^−1^ of the phages on the test strains divided by the PFU mL^−1^ on strain Vc2, which indicates no plaques formed. ^a^-no plaques formed.

Phage	Bacterial Strain (EOP)			
	Vc1	Vc2	Vc3	Vc4
KVP40	0.03 ± 0.01	1.0	155.3 ± 57.8	188.2 ± 60.7
φ919TP *cI*^-^	6.5 × 10^−5^ ± 9.01 × 10^−6^	1.0	-	-
φ919TP	- ^a^	1.0	-	-

## Data Availability

Not applicable.

## Supplementary Materials

The following are available online at https://www.mdpi.com/article/10.3390/v13122342/s1, Table S1: Bacterial strains, plasmids, and bacteriophage, Table S2: Oligonucleotides used in this study, Table S3: Comparative analysis of SNPs and Indels identified in the phage 919TP *cI*^-^ resistant mutants, Figure S1: Genomic structure of prophage genome 919TP. References [3,8,46] are cited in the Supplementary Materials.
