# Supplementary material for: Interactions between the Prophage 919TP and Its Vibrio cholerae Host: Implications of gmd Mutation for Phage Resistance, Cell Auto-Aggregation, and Motility
Source: Viruses. 2021 Nov 23;13(12):2342. doi: 10.3390/v13122342 (PMC8706939; doi:10.3390/v13122342)
Supplement: Supplementary file 1 [file viruses-13-02342-s001.zip › viruses-1433917-supplementary.pdf]

Table S1. Bacterial strains, plasmids and bacteriophage.

Name	Genotype or relevant markers
Escherichia coli	
S17-1	*thi pro hsdR hsdM⁺recA RP4-2-Tc::Mu-Km::Tn7 λpir* [8]
Vibrio cholerae	
Vc1	wt, *V. cholerae* O1 El Tor strain 919T [3]
Vc2	Δ919TP
Vc3	Vc2-919TP *cI*⁻ resistant strain
Vc4	Δ*gmd*
pDM4	Cm^r; suicide vector with an R6K origin (*pir* requiring) and *sacBR* genes from *Bacillus subtilis* [8]
pDM4919TP	Cm^r; pDM4 derivative containing 919TP fused in-frame
pDM4gmd	Cm^r; pDM4 derivative containing *gmd* fused in-frame
pDM4cI	Cmr; pDM4 derivative containing *cI* fused in-frame
919TP	*Siphoviridae*, isolated from Vc1 cell-free spent [3]
919TP *cI*⁻	*Siphoviridae*, lytic variant of phage 919TP
KVP40	*Myoviridae*, isolated from Japan seawater [46]

Table S2. Oligonucleotides used in this study.

Name	Primers (5'-3')
1	GCTGATGATTTCCGCATGGG
2	CGGCACAGACAGTGCCCAGC
3	CGTTGAAGCCGAGAGCGGCC
4	CGGCAATCGGGCCATTCATC
5	CCGCCATTTATGGCGACAAACCC
6	GGCAGCATCGACAATCGCC
919TP deletion	
919TP_1	TTTTCTAGAGGGGGGTACCACCTACCGCTCG
919TP_2	GATAAAGGTGGGGGTACTTTGAGAGAACCGGGGCTATTTGGTGGTAGCC
919TP_3	GCCCCGGTTCTCTCAAAGTACCCCCACCTTTATCTTTCCTACTTTTTCC
919TP_4	TTTCTCGAGCCGTCACGTCTTGCGCAGCACCG
cI deletion	
CI_1	TTTTCTAGACAGCGCAGCCAGTGTCCGTG
CI_2	CCCTAGCCAATTAAAAATTACAGTTGTCAATTGACGATCTAATATCTG
CI_3	CGTCAATTGACAACTGTAATTTTTAATTGGCTAGGGAATGAAAGATGACAGATAG
CI_4	TTTCTCGAGCCGGGGATTGGCGCACCTTG
qPCR	
flaC_F	GAACGCCTCTCATCAGGTAATC
flaC_R	CCACATCCAAACCACGAGAT
flaD_F	CGGTACATTCGCAACCAAATC
flaD_R	TACCTCCCATCAAAGCGTTATC
hfq_F	CTGCAAGGTCAGATCGAATCA
hfq_R	AGCAGGAACCACAGTAGAAATC

Table S3. Comparative analysis of SNPs and Indels identified in the phage 919TP *cI*$^-$ resistant mutants.

Chromosome	Position	Reference	Alteration	Effect	Impact	Gene
Vc1	32686	T	TC	frameshift_variant	HIGH	trkG
Vc1	100233	G	GC	frameshift_variant	HIGH	cc4
Vc1	184278	A	AG	frameshift_variant	HIGH	epsM_1
Vc1	197643	C	CG	frameshift_variant	HIGH	recG_2
Vc1	378854	T	TC	intergenic_region	MODIFIER	ptsO
Vc1	394742	G	GC	intergenic_region	MODIFIER	pyrB-argF
Vc1	433416	A	AC	frameshift_variant	HIGH	serA
Vc1	437308	C	CG	intergenic_region	MODIFIER	ubiH
Vc1	499117	T	TC	intergenic_region	MODIFIER	ampD-fldA_1
Vc1	585770	T	TG	frameshift_variant	HIGH	nupX_1
Vc1	588667	C	CG	intergenic_region	MODIFIER	deoA-deoB
Vc1	652122	A	AG	intergenic_region	MODIFIER	dinB-PROKKA_00619
Vc1	688760	C	CG	intergenic_region	MODIFIER	skp-lpxD
Vc1	823453	T	TG	frameshift_variant	HIGH	dapE_1
Vc1	914073	T	TC	intergenic_region	MODIFIER	cheW_1-ccmA
Vc1	914658	A	AG	frameshift_variant	HIGH	ccmA
Vc1	963867	A	AG	frameshift_variant	HIGH	PROKKA_00944
Vc1	1069599	T	TG	frameshift_variant	HIGH	PROKKA_01040
Vc1	1122494	A	AG	intergenic_region	MODIFIER	PROKKA_01080
Vc1	1270500	T	TG	frameshift_variant	HIGH	PROKKA_01218
Vc1	1538441	GAATAATTC	G	intergenic_region	MODIFIER	fabA
Vc1	1538561	C	CA	intergenic_region	MODIFIER	fabA
Vc1	1538601	C	CA	intergenic_region	MODIFIER	fabA
Vc1	1538606	C	CG	intergenic_region	MODIFIER	fabA
Vc1	1538613	AC	A	intergenic_region	MODIFIER	fabA
Vc1	1809152	A	AG	intergenic_region	MODIFIER	setA-btr_1
Vc1	1838595	G	GC	frameshift_variant	HIGH	cdd_1
Vc1	1909685	GC	G	frameshift_variant	HIGH	PROKKA_01781
Vc1	2185569	A	AC	intergenic_region	MODIFIER	pleD_3-ygdH_1
Vc1	2185636	T	TC	intergenic_region	MODIFIER	pleD_3-ygdH_1
Vc1	2185967	T	TC	frameshift_variant	HIGH	ygdH_1
Vc1	2190123	A	AC	frameshift_variant	HIGH	thiI_1
Vc1	2196162	C	CG	frameshift_variant	HIGH	dxs_1
Vc1	2229096	C	CA	intergenic_region	MODIFIER	pasT-smpB
Vc1	2230323	G	GC	intergenic_region	MODIFIER	smpB-intA_3
Vc1	2231421	G	GC	frameshift_variant	HIGH	intA_3
Vc1	2234249	T	TC	frameshift_variant	HIGH	PROKKA_02091
Vc1	2467731	T	TC	frameshift_variant	HIGH	greA_1
Vc1	2475046	C	CG	frameshift_variant	HIGH	cnrA_2
Vc1	2482039	A	AC	frameshift_variant	HIGH	rsmC_1
Vc1	2515704	C	CG	frameshift_variant	HIGH	hrpB_2
Vc1	2519088	T	TC	frameshift_variant	HIGH	PROKKA_02337
Vc1	2579580	T	TC	intergenic_region	MODIFIER	rpoS-nlpD
Vc1	2651682	G	GC	frameshift_variant	HIGH	pilT_1
Vc1	2879370	A	AG	intergenic_region	MODIFIER	ybdG
Vc1	2889666	T	TC	frameshift_variant	HIGH	PROKKA_02709
Vc1	2899167	CG	C	frameshift_variant	HIGH	gmd
Vc1	2900286	C	A	missense_variant	MODERATE	algC
Vc1	2932819	T	TC	intergenic_region	MODIFIER	murP
Vc1	2934409	G	GC	frameshift_variant	HIGH	murQ_2
Vc1	3019058	G	GC	intergenic_region	MODIFIER	atpH-atpF
Vc1	26506	T	TG	frameshift_variant	HIGH	cheR_2
Vc1	54635	A	AG	intergenic_region	MODIFIER	lrp_4-calB
Vc1	64530	T	TG	intergenic_region	MODIFIER	pctB_6
Vc1	72744	G	GC	intergenic_region	MODIFIER	yceM
Vc1	95420	T	TC	intergenic_region	MODIFIER	glgX-lamB
Vc1	98486	T	TG	frameshift_variant	HIGH,HIGH	PROKKA_02930
Vc1	106432	T	TC	intergenic_region	MODIFIER	alkA-ogt_2
Vc1	500776	G	T	missense_variant	MODERATE	PROKKA_03298
Vc1	816823	GGCGAAGAT	C	frameshift_variant	HIGH	ulaC
Vc1	980739	T	TC	frameshift_variant	HIGH	PROKKA_03777

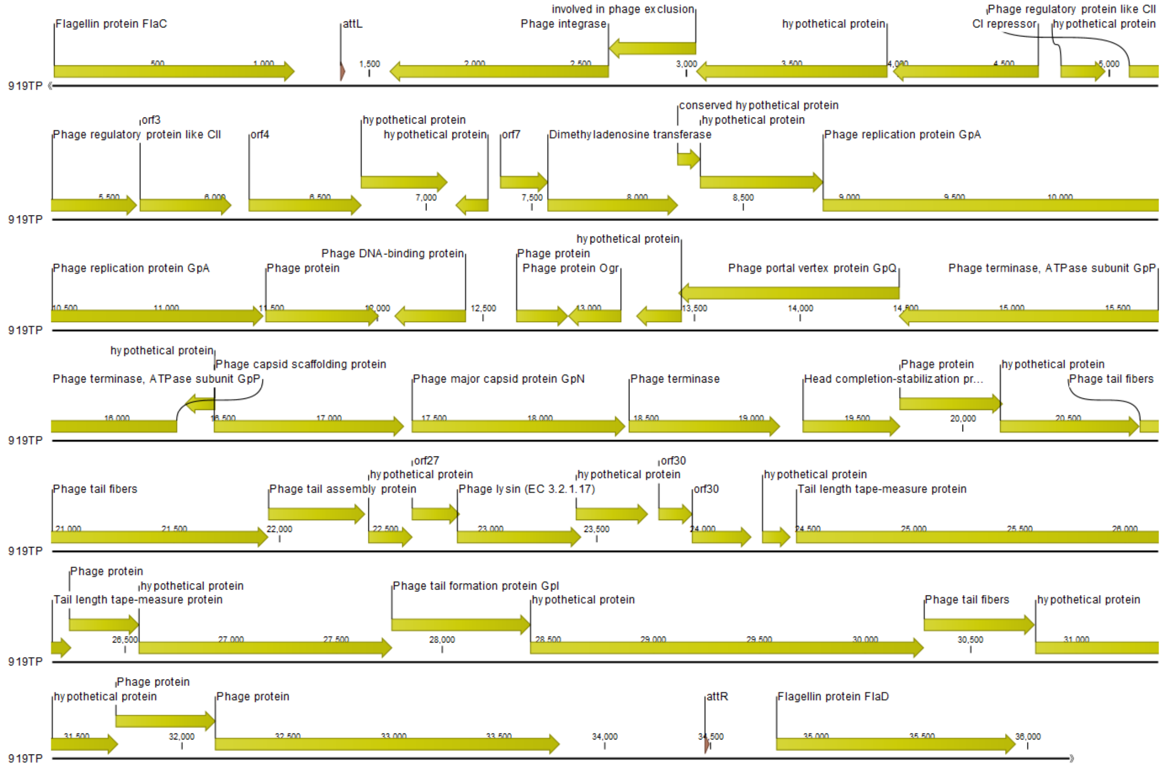

Figure S1. Genomic structure of prophage genome 919TP. The genome map was performed using the CLC Main Workbench, version 12.0 (CLC bio, Qiagen, Denmark). Arrows represent predicted ORFs, the direction of the arrow represents the direction of transcription.

References

3. Shen, X.; Zhang, J.; Xu, J.; Du, P.; Pang, B.; Li, J.; Kan, B.J.F.i.m. The resistance of Vibrio cholerae O1 El Tor strains to the typing phage 919TP, a member of K139 phage family. **2016**, *7*, 726.
8. Milton, D.L.; O'Toole, R.; Horstedt, P.; Wolf-Watz, H.J.J.o.b. Flagellin A is essential for the virulence of Vibrio anguillarum. **1996**, *178*, 1310-1319.
46. Matsuzaki, S.; Tanaka, S.; Koga, T.; Kawata, T.J.M.; immunology. A broad‐host‐range vibriophage, KVP40, isolated from sea water. **1992**, *36*, 93-97.
